# Amenorrhœa Cured by Electro-Galvanism

**Published:** 1844-11

**Authors:** 


*Case of Amenorrhœa cured by Electro-Galvanism.*—U. W., æt. 35, unmarried, rather delicate constitution; her menses had stopped by taking cold, some seven months before I first saw her. I tried all the usual remedies prescribed in such cases, but Idid not succeed in bringing about the desired effect. I had been using the Electro-Magnetic machine in some other cases with the happiest effects; and I therefore was induced to use it in this case, in which it succeeded most perfectly. I applied the buttons connected with the machine over the region of the womb, holding one on the lumbar region of the spine, and the other in front over the pubic region; using the negative and positive wires alternately to the spine and abdomen. I continued the remedy five days, from five to ten minutes each day, when her menses were fully re-established. I have used it in rheumatism with good effect.—*Ibid.*

